# A Study in a Regional Hospital of a Mid-Sized Spanish City Indicates a Major Increase in Infection/Colonization by Carbapenem-Resistant Bacteria, Coinciding with the COVID-19 Pandemic

**DOI:** 10.3390/antibiotics10091127

**Published:** 2021-09-18

**Authors:** Estefanía Cano-Martín, Inés Portillo-Calderón, Patricia Pérez-Palacios, José María Navarro-Marí, María Amelia Fernández-Sierra, José Gutiérrez-Fernández

**Affiliations:** 1 Department of Preventive Medicine and Public Health, Virgen de las Nieves University Hospital & ibs, Granada-Instituto de Investigación Biosanitaria de Granada, Avda. de las Fuerzas Armadas, 2, 18014 Granada, Spain; estefania.cano.sspa@juntadeandalucia.es (E.C.-M.); mamelia.fernandez.sspa@juntadeandalucia.es (M.A.F.-S.); 2 Unidad de Gestión Clínica de Enfermedades Infecciosas y Microbiología Clínica, Hospital Universitario Virgen Macarena & Instituto de Biomedicina de Sevilla (IBIs), Hospital Universitario Virgen Macarena/CSIC/Universidad de Sevilla, 41020 Sevilla, Spain; inesm.portillo.sspa@juntadeandalucia.es (I.P.-C.); pperez5@us.es (P.P.-P.); 3 Laboratory of Microbiology, Virgen de las Nieves University Hospital. & ibs, Granada-Instituto de Investigación Biosanitaria de Granada, Avda. de las Fuerzas Armadas, 2, 18014 Granada, Spain; josem.navarro.sspa@juntadeandalucia.es; 4 Department of Microbiology, School of Medicine, University of Granada & ibs, Granada-Instituto de Investigación Biosanitaria de Granada, Avda. de la Investigación, 11, 18016 Granada, Spain

**Keywords:** carbapenems resistance, carbapenemases, Gram-negative bacteria, infection, colonization, COVID-19

## Abstract

Bacterial resistance to antibiotics has proven difficult to control over the past few decades. The large group of multidrug-resistant bacteria includes carbapenemase-producing bacteria (CPB), for which limited therapeutic options and infection control measures are available. Furthermore, carbapenemases associate with high-risk clones that are defined by the sequence type (ST) to which each bacterium belongs. The objectives of this cross-sectional and retrospective study were to describe the CPB population isolated in a third-level hospital in Southern Spain between 2015 and 2020 and to establish the relationship between the ST and the epidemiological situation defined by the hospital. CPB were microbiologically studied in all rectal and pharyngeal swabs and clinical samples received between January 2015 and December 2020, characterizing isolates using MicroScan and mass spectrometry. Carbapenemases were detected by PCR and Sanger sequencing, and STs were assigned by multilocus sequence typing (MLST). Isolates were genetically related by pulsed-field gel electrophoresis using Xbal, Spel, or Apal enzymes. The episodes in which each CPB was isolated were recorded and classified as involved or non-involved in an outbreak. There were 320 episodes with CPB during the study period: 18 with *K. pneumoniae*, 14 with *Klebisella oxytoca*, 9 with *Citrobacter freundii*, 11 with *Escherichia coli*, 46 with *Enterobacter cloacae*, 70 with *Acinetobacter baumannii*, and 52 with *Pseudomonas aeruginosa*. The carbapenemase groups detected were OXA, VIM, KPC, and NDM with various subgroups. Synchronous relationships were notified between episodes of *K. pneumoniae* and outbreaks for ST15, ST258, ST307, and ST45, but not for the other CPB. There was a major increase in infections with CPB over the years, most notably during 2020, coinciding with the COVID-19 pandemic. This study highlights the usefulness of gene sequencing techniques to control the spread of these microorganisms, especially in healthcare centers. These techniques offer faster results, and a reduction in their cost may make their real-time application more feasible. The combination of epidemiological data with real-time molecular sequencing techniques can provide a major advance in the transmission control of these CPB and in the management of infected patients. Real-time sequencing is essential to increase precision and thereby control outbreaks and target infection prevention measures in a more effective manner.

## 1. Introduction

There has been an alarming rise in bacterial resistance to antibiotics over the past two decades, representing a “silent pandemic” that appears unstoppable. This resistance has become especially frequent in healthcare-related infections (HCRIs) worldwide [1,2,3]. However, there has also been an increase in the emergence and transmission of multidrug-resistant bacteria in the community setting over the past few years, including residential facilities and care homes. The more frequent hospitalization of these residents has favored exchanges between hospital and community, increasing the capacity to spread resistant bacteria in each setting [3]. The World Health Organization (WHO) has declared infection by these microorganisms as an emerging disease that poses a major public health threat worldwide [1,2,3].

The large group of carbapenem-resistant bacteria includes carbapenemase-producing bacteria (CPB), for which there are limited therapeutic options, and infection control measures have proven ineffective to prevent the dissemination of these bacteria to date [4]. The WHO list of global priority pathogens includes carbapenem-resistant *Acinetobacter baumannii* (CRAb) and *Pseudomonas aeruginosa* (CRPa) and carbapenem-producing *Enterobacteriaceae* (CPE) [2]. The most frequently observed carbapenem-resistance mechanism is the production of carbapenemases [5]. Control of these infections is further hampered by the horizontal transmission of these enzymes via plasmids among different *Enterobacteriaceae* [4].

In 2018, one-third of *Acinetobacter* spp. isolates obtained in the European Union (EU) were resistant [6], and numerous outbreaks increased morbidity and mortality rates, mainly in intensive care units (ICUs). A worldwide increase was observed in the frequency of patients colonized or infected by CPE, which was above the EU mean in Spain [5]. The European Center for Disease Control (ECDC) reported an increase in combined resistances in *Escherichia coli* and *Klebsiella pneumoniae* over the past few years [3]. *K. pneumoniae* is the most prevalent CPE [2] and responsible for the majority of outbreaks in healthcare centers [7]. CPE infections were first detected in Spain in 2005, and their number had multiplied 16-fold by 2019 [8].

In addition, carbapenemases are enzymes that associate with certain high-risk clones [8] that are defined by the sequence type (ST) to which each bacterium belongs. The STs most frequently associated with carbapenemases in *K. pneumoniae* are ST258, which is predominant worldwide [8], and ST307, which is also associated with a higher mortality [9]. In Germany, it was reported that *E. coli* ST131 is a high-risk clone that should be monitored very closely [10], alongside ST38, located in various European countries [10]. In Spain, the STs most frequently associated with carbapenemases are ST11, 13, 15, 16, 101, 147, 340, 384, 388, 405, 437, 464, 512, 846 and 1235 [8].

Carbapenemases can be divided into metallocarbapenemases (zinc-dependent class B) and non-metallocarbapenemases (zinc-independent classes A, C, and D) [11]. The first carbapenemase detected was in class A *K. pneumoniae* [11], and some carbapenemases appear more frequently than others. The first carbapenemase-producing isolates detected in Spain belonged to the KPC group [8]. OXA-48 group carbapenemases are currently the most abundant, mainly among *Klebsiella* spp. and *Enterobacter* spp. [5], and are the most common resistance mechanism among CPEs in Spain [5]. Other carbapenemases frequently reported in Spain are VIM-1, KPC-2, IMP, and NDM-1 [8].

Since 2020, a rise in CPB resistance in our setting coincided with the COVID-19 pandemic, which has been accompanied by populations at risk of severe complications and long-term sequelae and by longer hospital stays and the prescription of an elevated amount of antibiotics. These factors have led to an elevated risk of nosocomial CBP infections, as recently reported [12,13,14]. In addition, COVID-19 was especially prevalent in our hospital catchment area from 2020, and the patients frequently required hospitalization, as recorded by the Andalusian Health Service (https://www.juntadeandalucia.es/institutodeestadisticaycartografia/salud/datosSanitarios.html (accessed on 1 July 2021); and https://www.sspa.juntadeandalucia.es/servicioandaluzdesalud/todas-noticia/informacion-sobre-el-numero-de-casos-de-coronavirus-511?utm_source=servicioandaluzdesalud&utm_campaign=Boletin%20Novedades&utm_medium=mail&utm_content=20210901&utm_term=Informacion%20sobre%20el%20numero%20de%20casos%20de%20coronavirus (accessed on 1 July 2021).

The objectives of this study were to describe the CPB population isolated in a third-level hospital between 2015 and 2020 and to establish the relationship between STs and the epidemiological situation as defined by the hospital.

## 2. Material and Methods

This retrospective cross-sectional study included adult patients admitted to the Departments of Internal Medicine and its Specialties, ICUs (general and cardiac), and the Department of General Surgery and its Specialties of the Virgen de las Nieves University Hospital in Granada (Spain). This hospital provides specialized care to a population of around 331,220 inhabitants. No exclusion criteria were applied, except for repeat microbiological studies of the same episode. For the colonization study, the presence of CPB was studied in rectal (RS) and pharyngeal (PS) swabs received by the Clinical Microbiology Laboratory between 1 January 2015 and 31 December 2020 (5415 RS and 1034 PS for 3107 episodes studied before 2020 and 2308 during 2020). For the study of “possible infection episodes”, clinical samples from different localizations were studied by applying standard clinical microbiology procedures, detecting CPB as previously described [15]. In brief, samples were seeded on selective culture medium CHROMID^®^ ESBL (BioMérieux, Marcy-l’Étoile, France) and incubated at 37 °C in aerobiosis for 48 h. Isolates were identified by using the MicroScan system (Beckman Coulter, Brea, CA, USA) and mass spectrometry (Maldi-Tof ^®^, Brucker Daltonik GmbH, Bremen, Germany). Resistance was characterized with the MicroScan system, using currently available Neg Combo panels, and interpreted according to the clinical cutoff points defined by the European Committee on Antimicrobial Susceptibility Testing (EUCAST) [16]. Carbapenemase was detected using the colorimetric Neo-Rapid CARB Kit ^®^ (Rosco Diagnostica A/S, Taastrup, Denmark) and immunochromatography (NG5-Test Carba, NG Biotech, Guipry-France to detect KPC, NDM, VIM, IMP, and OXA-48-like enzymes, and K-Set, Coris BioConcept, Gembloux, Belgium to detect OXA-23) in isolates meeting EUCAST cutoff point criteria for CPB screening. In parallel, isolates identified in swab and clinical samples were sent to the reference laboratory for molecular typing of nosocomial pathogens and genotypic detection of antimicrobial resistance mechanisms of interest under the regional Integrated Program for the Prevention and Control of Healthcare-related Infections and Appropriate Utilization of Antibiotics (acronym in Spanish, PIRASOA) led by the Microbiology Department of Virgen Macarena Hospital in Seville.

### 2.1. Microbiological Study of Carbapenemase-Producing Bacteria (CPB) under the PIRASOA Program

The susceptibility to ertapenem, imipenem, and meropenem was investigated by disk diffusion in Mueller Hinton agar, using EUCAST clinical cutoff points to interpret the results [16]. Carbapenemase activity inhibition [17] was studied by disk diffusion using meropenem, meropenem/boronic acid, meropenem/dipicolinic acid, and meropenem/cloxacillin disks as well as a temocillin disk (Rosco Diagnostica, Taastrup, Denmark). Carbapenemase and MLST genes were studied by PCR using specific primers and Sanger sequencing until 2018 and subsequently massive sequencing (Illumina Inc., San Diego, CA, USA). Sequences were analyzed with CLC Genomics Workbench, v10 software (Qiagen Iberia, Las Rozas de Madrid, Madrid, Spain). Determinants of resistance were detected using ResFinder (https://cge.cbs.dtu.dk/services/ResFinder) (accessed on 1 July 2021). and CARD (https://card.mcmaster.ca/) (accessed on 1 July 2021). databases, and MLST was identified using the MLST finder 2.0 database (https://cge.cbs.dtu.dk/services/MLST) (accessed on 1 July 2021). Clonal relationships among isolates were evaluated by pulsed-field gel electrophoresis (PFGE). Complete chromosomal DNA digestion in agarose gel was performed with XbaI (*Enterobacterales*), SpeI (*Pseudomonas* spp. and *Stenotrophomonas*), and ApaI (*Acinetobacter* spp.) according to the species. The resulting restriction fragments were separated in the CHIEF DR-II system (Bio-Rad Laboratories, Alcobendas, Madrid, Spain) with 1% agarose gel. The gels were subsequently stained with ethidium bromide, illuminated with ultraviolet light, and photographed in an automatic Gel Logic 200 Imaging System (Kodak, Rochester, NY, USA).

The conversion, normalization, and analysis of band patterns were performed using Bionumerics 7.6 software (AppliedMaths, Jollyville Rd., Austin, TX, USA), analyzing the patterns as previously described. Band position tolerance and optimization were set at 1%. An unweighted pair group method with arithmetic mean (UPGMA) was employed to generate a dendrogram and the Dice coefficient was used to measure genetic similarity among isolates. PFGE patterns with ≥90.0% similarity were considered in the same group as closely related isolates.

### 2.2. Epidemiological Study

Episodes in which each carbapenemase-producing microorganism was isolated were recoded to avoid repetition with the same patient. An episode was defined as each hospital stay in which one or several different carbapenemase-producing microorganisms were isolated, only considering the first isolate of each microorganism during the hospital stay for infection or colonization study. When there were multiple isolated of the same microorganism during the same episode, one was selected according to the following criteria and order:

1. Isolate described in a PIRASOA report;
2. Isolate corresponding to infection study;
3. Isolate not reported in infection study, corresponding to colonization study.

The PIRASOA program describes the ST of the microorganism and its genetic similarity to other microorganisms of the same species from any hospital in Andalusia. It also reports whether the relationship with other microorganisms was recent or derived from a common transmission focus. It was determined whether a CPB had a synchronous (SR) or asynchronous relationship (AR) with others of the same species when the following criteria were met: (1) they belong to the same clone of an ST, (2) patients coincided in their hospital stay, and/or (3) the genetic study indicates direct transmission among patients and/or very recent exposure to a common reservoir. An SR was considered when the first criterion and one other criterion were met and an AR when only one criterion was met.

It was also recorded whether the CPB was involved or not in an outbreak of HCRI, defined by two or more cases of HCRI due to the same microorganism and associated in space and time with suspicion of an epidemiological link. The emergence of a single case of HCRI by a new, or unknown, or re-emergent infectious agent of mandatory declaration was considered an outbreak of nosocomial infection, henceforth “nosocomial outbreak” [18].

### 2.3. Influence of COVID-19 Infection during 2020

Data were compared between 2019 and 2020 on tested patients with a positive result for COVID-19 (*n* = 178 episodes), including their age, sex, length of hospital stay, and in-hospital consumption of imipenem (IP), meropenem (MP), and/or piperacillin–tazobactam (PTZ) as daily dose per 1000 stays (DDD/1000 stays).

### 2.4. Statistical Study

The involvement of episodes in which an SR was reported in a declared nosocomial outbreak was examined by constructing contingency tables and applying Fisher’s exact test. The comparison between sexes was analyzed in the same way. The comparison by age was studied with the Student’s *t*-test after establishing the normality of data distribution with the Kolmogorov–Smirnov test. IBM SPSS Statistics v. 19 was used for data analyses. *p* < 0.05 was considered significant.

## 3. Results

Between 1 January 2015 and 31 December 2020, 320 episodes of CPB were identified: 118 episodes of *K. pneumoniae*, 14 of *Klebisella oxytoca*, 9 of *Citrobacter freundii*, 11 of *E. coli*, 46 of *Enterobacter cloacae*, 70 of *A. baumannii*, and 52 of *P. aeruginosa*. Among these, 39 episodes were excluded because there was no definitive PIRASOA report and four because the clinical history of the patient was missing from the Andalusian Health Service database. Among the 43 episodes (13.4%) lost to the study, 34.9% involved *K. pneumoniae*, 6.9% *K. oxytoca*, 4.6% *C. freundii*, 6.9% *E. coli*, 2.3% *E. cloacae*, 13.9% *A. baumannii*, and 30.2% *P. aeruginosa*. Out of the final sample of 277 episodes, 103 involved *K. pneumoniae*, 11 *K. oxytoca*, 7 *C. freundii*, 8 *E. coli*, 45 *E. cloacae*, 64 *A. baumannii*, and 39 *P. aeruginosa*.

### 3.1. Description of Episodes

The most frequently isolated CPB in the studied episodes was *K. pneumoniae* (37.19% of total episodes), followed by *A. baumannii* (23.10%) and *E. cloacae* (16.25%) (Table 1). *K. pneumoniae* was detected in more numerous episodes every year except for 2018, when more episodes with *P. aeruginosa* were detected, and 2019, when there were more episodes with *A. baumannii*. Over the six-year study period, episodes with CPB were most frequent in 2020 (37.54%). There has been an increase in *A. baumannii* in episodes over the past two years and an increase in *E. cloacae* over the past year. The number of episodes each year rose from 6 in 2015 (2.17% of total episodes) to 123 in 2020 (37.54%). Globally, there were 156 episodes of infection and 121 of colonization. However, as depicted in Table 1, infection episodes were much more frequent than colonization episodes in 2016 and 2017, there was a similar frequency of infection and colonization episodes in 2018 and 2019, and colonization episodes (57) were much more frequent in 2020 (47).

The carbapenemase groups detected in this study were OXA, VIM, KPC, and NDM with their subgroups (Table 2). Table 3 exhibits the relationships between ST and carbapenemase for each species. There were four peak episodes with *K. pneumoniae* of ST258, ST307, ST15, and ST45, mainly associated with KPC-3, OXA-48, NDM-5, and OXA-48, respectively. There were fewer episodes with *K. oxytoca* and *C. freundii*, and only four STs were found in *K. oxytoca*, highlighting the association of ST36 with VIM-1, and six STs in *C. freundii*, highlighting the production of OXA-48 in three of the STs. Table 3 highlights the production of OXA-48 in *E. coli* with ST58, ST69, ST405, and ST648. However, the most frequent carbapenemase in *E. cloacae* was VIM-1, closely related to ST78, with OXA-48 being the second most frequent. Notably, the production of OXA-23 was associated with ST2 in *A. baumannii.* A wide variety of carbapenemases was detected in *P. aeruginosa*, highlighting the relationship of IMP-8 with ST348, the most frequent ST. ST175 and ST253 were less frequently detected, mainly associated with OXA-50 and IMP-16, respectively.

### 3.2. Description of the Association among ST, Type of Relationship, and Involvement in a Nosocomial Outbreak of Infection with CPB

This association was more frequent in *K. pneumoniae*, *E. cloacae*, *A. baumannii*, and *P. aeruginosa* (Table 4). In *K. pneumoniae*, SRs were only reported for ST15, ST258, ST307, and ST45, the STs with episodes included in outbreaks. ARs alone were reported for the other STs detected, none of which had more than three episodes. An association was found between the presence of an SR in episodes and involvement in an outbreak (*p* = 0.000007299). In *E. cloacae*, PIRASOA reported an SR in only two of the eight STs detected (ST114 and ST78), but only ST114 was involved in an outbreak, and the association between the presence of an SR and involvement in an outbreak was just short of statistical significance (*p* = 0.06829). In *A. baumannii*, only two STs (ST1 and ST2) were found, being ST1 in all episodes except for one; *A. baumannii* had an SR with others in half of the episodes and an AR in the remaining half. No significant association was found (*p* = 1) between the presence of an SR and involvement in an outbreak. Finally, in *P. aeruginosa*, six STS were detected and four of these were SRs: ST175, ST253, ST348, and ST845, but only ST253 was involved in an outbreak. No significant association was found (*p* = 1) between the presence of an SR and involvement in an outbreak.

As shown in Table 5, episodes with SR did not correspond to episodes involved in an outbreak, and episodes with AR were even involved in outbreaks in some cases.

### 3.3. Influence of Infection with COVID-19 during 2020

The absolute number of CPB infections was higher during 2020 than during 2019, attributable to a marked rise in the number of samples studied rather than a higher percentage of positive results (4.5% in 2020 vs. 5.6% in 2019). Table 6 compares clinical data of patients testing during 2019 (74) and 2020 (104), positive for COVID-19 infection, including age (*p* = 0.014), sex (not significant), hospital stay, hospital occupation, and prescriptions of PTZ, IMP, and PTZ (daily dose per 1000 stays).

## 4. Discussion

This study describes carbapenemase-producing microorganisms isolated over the past six years in the microbiological laboratory of a tertiary hospital in Southern Spain. The main findings were an increasingly elevated transmission of these bacteria in both hospital and community settings and a major rise in the detection of CPB.

The capacity to detect CPB and their resistances has significantly improved through technological advances and the rigorous implementation of screening and control protocols. However, the upsurge of CPB cases in 2020 can be attributed at least in part to the COVID-19 pandemic, which led to a major rise in the number of studied episodes due to an increase in hospital stays, among other reasons. The resulting overload of hospital departments hampered the application of infection prevention measures, and there was an increased administration of antibiotics (often inappropriate) to prevent and treat bacterial over-infection in patients with pneumonia [19]. The inappropriate use of antibiotics is one cause of the increasingly frequent emergence of resistance to carbapenem, among other antibiotics. However, improvements in the diagnostic capabilities of microbiology laboratories over time should also be taken into account, which would also influence an increase in the detection of infectious episodes.

Four groups of carbapenemases (OXA, VIM, KPC, and NDM) and corresponding subgroups were detected, all previously reported in Spain. Some were found in more than one microorganism species (OXA-48, IMP-8, VIM-1, VIM-63, and KPC-3) and the others were detected in a single species. The most abundant subgroup was OXA-48 (in 29.79% of episodes), which is also the most prevalent subgroup worldwide [5], followed by VIM-1 (17.12%), OXA-23 (16.44%), and KPC-3 (12.33%). The most frequently isolated bacteria in these episodes was *K. pneumoniae*, one of the most widely disseminated CPB worldwide and largely responsible for the alarm caused by CPB, given the limited therapeutic options and high mortality rates [7].

The relationships between STs and carbapenemases were explored in the detected CPB, followed by a search for ST–carbapenemase combinations that have been reported in Spain for each CPB, highlighting isolates of: *K. pneumoniae* ST15-OXA-48 [20,21], ST258-KPC-3 [8,22], ST307-OXA-48 [23,24], and ST512-KPC-3 [23,25,26,27]; *K. oxytoca* ST36-VIM 1 [28]; *E. coli* ST58-OXA-48 [29]; *E. cloacae* ST114-OXA-48 [30]; *A. baumannii* ST1-OXA-23 [31]; and *P. aeruginosa* ST175-VIM-2 [32,33].

No other combinations were found, either because they were not previously detected in a Spanish hospital or were detected but not published. CPB associated with STs or carbapenemases were found, but no relationship was observed between STs and carbapenemase.

Although not as notorious as *K. pneumoniae*, the other CPB described in this study form part of a group of multidrug-resistant microorganisms that pose a major public health threat [34] and show increased diversity over time, as reported in a Portuguese study [4]. The frequency of their detection is rising among colonized and infected hospital patients, despite the implementation of infection prevention and control measures and routine colonization screenings [4]. There is also a trend towards more frequent nosocomial outbreaks, especially of *K. pneumoniae* [7], and towards their longer persistence as colonizers of hospitalized patients and in the hospital setting. This creates “silent” reservoirs and carriers of CPB over prolonged time periods, hampering control of their dissemination [7,11]. The comprehensive and exhaustive implementation of available control measures is essential. In our setting, these include the surveillance and follow-up of HCRIs and nosocomial outbreaks and active communication from the microbiology departments on microorganisms of concern.

We highlight the importance of genetic sequencing techniques for controlling the spread of these microorganisms, given the speed with which results are obtained. A reduction in their cost may allow their real-time performance to be more widely available at health centers. Data obtained on the STs and carbapenemase subgroup of a CPB indicate its pathogenicity, dissemination capacity, local prevalence, therapeutic options, and responses to treatment and can also yield information on changes in the resistances of these bacteria [4,7]. These techniques can be highly effective for the detection and control of nosocomial outbreaks. When only the species and antibiogram of microorganisms are identified but not their ST, it is not known whether multiple unrelated STs might be involved, and this question would be resolved by real-time molecular analysis. Without this information, an outbreak is declared when there appear to be two or more cases of hospital-acquired infection. Accordingly, an outbreak is declared in the hospital when there are two or more patients with the infection who shared hospital space and/or staff/equipment at some time since their admission (epidemiological link). If cases continue to emerge among patients successively admitted at different times to the same unit, the possibility of a common reservoir is investigated to establish the epidemiological link, i.e., an SR. The association observed between the presence of an SR and involvement in outbreaks suggests that the epidemiological criteria for declaring an outbreak were correct because relationships were observed among microorganisms that were subsequently found to have the same lineage. However, it cannot be assumed that CPB with identical STs are involved in the same chain of transmission without considering the epidemiological evidence [35].

In conclusion, there has been a major increase in infections with CPB over the years, especially during 2020, coinciding with the COVID-19 pandemic. The combination of epidemiological data with real-time molecular sequencing data represents a major advance in the control of CPB transmission and the management of infected patients. The increased precision offered by molecular sequencing techniques and the possibility of their real-time performance can contribute to a greater control of nosocomial outbreaks and a more effective targeting of infection prevention measures.

## Figures and Tables

**Table 1:** Colonization/infection episodes of each carbapenemase-producing bacteria in each year.

	Years/Episodes													
Microorganisms	2015		2016		2017		2018		2019		2020		Total	
	Infection	Colonization	Infection	Colonization	Infection	Colonization	Infection	Colonization	Infection	Colonization	Infection	Colonization	Infection	Colonization
*K. pneumoniae*	1	3	16	5	10	2	6	2	12	5	21	20	66	37
*K. oxytoca*	0	0	3	0	2	0	2	2	0	1	0	0	7	4
*C. freundii*	0	0	0	0	0	0	0	0	0	2	2	3	2	5
*E. coli*	0	0	0	0	1	0	1	1	0	1	2	2	4	4
*E. cloacae*	0	2	4	0	3	1	6	2	4	2	12	9	29	16
*A. baumannii*	0	0	0	0	0	0	2	2	17	17	8	18	27	37
*P. aeruginosa*	0	0	0	0	7	0	6	6	6	7	2	5	21	18
Total	1 (16.7%)	5 (83.3%)	23 (82.1%)	5 (17.9%)	23 (85.2%)	4 (14.8%)	23 (60.5%)	15 (39.5%)	39 (52.7%)	35 (47.3%)	47 (45.2%)	57 (54.8%)	156 (56.3%)	121 (43.7%)

**Table 2:** Subgroups of carbapenemases detected in each carbapenemase-producing bacteria (CPB).

Carbapenemase Types																	
	OXA							VIM			IMP			KPC		NDM	Total
Microorganisms	OXA-1	OXA-23	OXA-48	OXA-50	OXA-58	OXA-244	OXA-245	VIM-1	VIM-2	VIM-63	IMP-8	IMP-16	IMP-23	KPC-2	KPC-3	NDM-5	
*K. pneumoniae*	1		56				1	4			1			1	32	8	102
*K. oxytoca*			1					8						2			11
*C. freundii* *			4					2		1				1	2		10
*E. coli*			5			1		2									8
*E. cloacae*			16					28			1						45
*A. baumannii*		47			17												64
*P. aeruginosa* *				1				2	3	1	14	10	9				40
Total (%)	1 (0.36%)	47 (16.79%)	82 (29.99%)	1 (0.36%)	17 (6.07%)	1 (0.36%)	1 (0.36%)	46 (16.43%)	3 (1.07%)	2 (0.71%)	16 (5.71%)	10 (3.57%)	9 (3.21%)	4 (1.43%)	32 (11.43%)	8 (2.86%)	280 (100%)

* CPB in which more than one carbapenemase is detected in an episode.

**Table 3:** Description in the microorganisms of carbapenemase and sequence types.

SEQUENCIO-TYPES	CARBAPENEMASE TYPES																
	KPC-2	KPC-3	0XA-1	OXA-23	0XA-48	0XA-50	0XA-58	0XA-244	0XA-245	VIM-1	VIM-2	VIM-63	IMP-8	IMP-16	IMP-23	NDM-5	Not Available
**ST1**									**1**								
**ST1**				**1**													
**ST2**				**46**			**17**										
**ST10**								**1**									
**ST11**			**1**							**1**							
ST15	**1**				**3**											**8**	
**ST18**		**2**															
**ST22**					**2**												
**ST24**													**1**				
**ST25**										**1**							
**ST36**										**9**							
**ST37**																	**1**
**ST45**					**11**												
**ST50**					**1**					**1**							
**ST58**					**1**												
**ST69**					**1**												
**ST78**										**22**							
**ST90**										**4**							
**ST108**					**1**												
**ST111**										**1**							
**ST114**					**13**												
**ST128**										**1**							
**ST145**										**1**							
**ST147**					**1**												
**ST170**	**2**																
**ST175**	**1**											**1**					
**ST175**											**2**				**9**		
**ST214**					**1**												
**ST238**					**1**							**1**					
**ST253**						**1**								**10**			
**ST258**		**29**															
**ST277**											**1**						
**ST307**					**29**												
**ST307/cgST3303**					**3**												
**ST307/cgST5556**					**1**												
**ST321**													**1**				
**ST348**													**14**				
**ST392**					**1**												
**ST405/cgST5158**					**3**												
**ST405**					**2**												
**ST512**		**1**															
**ST513**					**1**												
**ST525**										**1**							
**ST648**					**1**												
**ST845**										**2**							
**ST896**					**3**												
**ST1262**					1												
**ST1774**					**1**												
**ST2242**												**1**					
**ST8327**										**1**							
**Not available**										**1**							
	***K. oxytoca***																
	***E. coli***																
	***E. cloacae***																
	***A. baumannii***																
	***K. pneumoniae***																
	***C. freundii***																
	***P. aeruginosa***																

**Table 4:** Relationship between sequence types of each CPB, type of relationship, and involvement in a nosocomial outbreak.

***K. pneumoniae***	**Sequence Type**	**AR**	**SR**	**Total**	**OB NO**	**OB YES**	**Total**
	ST1	1	0	1	1	0	1
	ST11	2	0	2	2	0	2
	ST128	1	0	1	1	0	1
	ST147	1	0	1	1	0	1
	ST15	4	8	12	6	6	12
	ST1774	1	0	1	1	0	1
	ST25	1	0	1	1	0	1
	ST258	8	21	29	26	3	29
	ST307	8	21	29	20	9	29
	ST307/cgST3303	3	0	3	3	0	3
	ST307/cgST5556	1	0	1	1	0	1
	ST321	1	0	1	1	0	1
	ST37	1	0	1	1	0	1
	ST392	1	0	1	1	0	1
	ST405/cgST5158	3	0	3	3	0	3
	ST45	4	7	11	7	4	11
	ST512	1	0	1	1	0	1
	ST525	1	0	1	1	0	1
	ST896	3	0	3	3	0	3
	Total	46	57	103	81	22	103
***E. cloacae***	ST108	1		1	1		1
	ST111	1		1	1		1
	ST114	3	10	13	10	3	13
	ST1262	1		1	1		1
	ST24	1		1	1		1
	ST50	2		2	2		2
	ST78	13	9	22	22		22
	ST90	4		4	4		4
	Total	26	19	45	42	3	45
***A.baumannii***	ST1	1	0	1	1	0	1
	ST2	31	32	63	45	18	63
	Total	32	32	64	46	18	64
***P. aeruginosa***	ST175	3	8	11	11		11
	ST2242	1		1	1		1
	ST253	4	6	10	4	6	10
	ST277	1		1	1		1
	ST348	1	13	14	14		14
	ST845	1	1	2	2		2
	Total	11	28	39	33	6	39

Type of relationship: synchronous (SR) or asynchronous relationship (AR). Outbreak: OB.

**Table 5:** Comparison between identified SRs and those involved in a nosocomial outbreak of CPB.

CPBs	SR	OB YES	AR	OB YES
*K. pneumoniae*	57	21	46	1
*K. oxytoca*	4	0	7	0
*C. freundii*	2	0	5	0
*E. cloacae*	19	3	26	0
*A. baumannii*	32	12	32	6
*P. aeruginosa*	28	4	11	2

Type of relationship: synchronous (SR) or asynchronous relationship (AR). Outbreak: OB.

**Table 6:** Clinical data for episodes of carbapenemase-producing bacteria.

Parameters	2019	2020
Age, years	63.36	57.48
Standard deviation	16.601	17.254
Males, %	65	60
Females, %	35	40
Hospital stay, days	7.75	8.79
Hospital occupation, %	77.56	77.24
Piperacillin–tazobactam *	65.13	66.88
Imipenem *	6.71	5.13
Meropenem *	47.45	61.21

* daily dose per 1000 stays (DDD/1000).

## Data Availability

Not applicable.
